# Identification of Genetic Markers for the Detection of *Bacillus thuringiensis* Strains of Interest for Food Safety

**DOI:** 10.3390/foods11233924

**Published:** 2022-12-05

**Authors:** Arnaud Fichant, Arnaud Felten, Armel Gallet, Olivier Firmesse, Mathilde Bonis

**Affiliations:** 1 Laboratory for Food Safety, University Paris-Est, French Agency for Food, Environmental and Occupational Health & Safety (ANSES), 94700 Maisons-Alfort, France; 2 Université Côte d’Azur, CNRS, INRAE, ISA, France; 3 Ploufragan-Plouzané-Niort Laboratory, Viral Genetics and Biosafety Unit, French Agency for Food, Environmental and Occupational Health & Safety (ANSES), 22440 Ploufragan, France

**Keywords:** *Bacillus thuringiensis*, *Bacillus cereus*, foodborne outbreak, genome-wide association study

## Abstract

*Bacillus thuringiensis* (Bt), belonging to the *Bacillus cereus* (Bc) group, is commonly used as a biopesticide worldwide due to its ability to produce insecticidal crystals during sporulation. The use of Bt, especially subspecies *aizawai* and *kurstaki*, to control pests such as Lepidoptera, generally involves spraying mixtures containing spores and crystals on crops intended for human consumption. Recent studies have suggested that the consumption of commercial Bt strains may be responsible for foodborne outbreaks (FBOs). However, its genetic proximity to Bc strains has hindered the development of routine tests to discriminate Bt from other Bc, especially *Bacillus cereus sensu stricto* (Bc ss), well known for its involvement in FBOs. Here, to develop tools for the detection and the discrimination of Bt in food, we carried out a genome-wide association study (GWAS) on 286 complete genomes of Bc group strains to identify and validate in silico new molecular markers specific to different Bt subtypes. The analyses led to the determination and the in silico validation of 128 molecular markers specific to Bt, its subspecies *aizawai*, *kurstaki* and four previously described proximity clusters associated with these subspecies. We developed a command line tool based on a 14-marker workflow, to carry out a computational search for Bt-related markers from a putative Bc genome, thereby facilitating the detection of Bt of interest for food safety, especially in the context of FBOs.

## 1. Introduction

*Bacillus cereus sensu lato*, or the *Bacillus cereus* group (Bc), is composed of Gram-positive, facultative anaerobic, spore-forming, ubiquitous bacteria. This group comprises at least nine species: *B. anthracis*, *B. cereus sensu stricto* (Bc s.s.), *B. thuringiensis* (Bt), *B. mycoides*, *B. pseudomycoides*, *B. weihenstephanensis*, *B. cytotoxicus*, *B. toyonensis* and *B. wiedmannii* [1]. Furthermore, Bc members have very similar characteristics and highly conserved genomes [2]. As a result, the taxonomic classification within the group is regularly revised, particularly due to the widespread use of whole-genome sequencing (WGS) which has led to a higher resolution for the definition of new species [3,4].

An large number of virulence genes can be found in the Bc group, causing the occurrence of foodborne outbreaks (FBOs). According to the European Food Safety Authority (EFSA), in 2019, Bc represented the leading cause of FBOs due to bacterial toxins in Europe [5]. Bc can cause gastrointestinal disorders, such as diarrhea, vomiting, abdominal pain, or a combination thereof. The diarrhea is due to the ingestion of bacteria that produce enterotoxins, including three major pore-forming toxins; the non-hemolytic enterotoxin (Nhe) is found in approximately 85% to 100% of Bc strains, and hemolysin BL (Hbl) and cytotoxin K (CytK) are found in approximately 40 to 70% [6]. In contrast, the cereulide toxin, produced in food by some vegetative Bc, can lead to emetic syndromes, which predominantly cause vomiting. The *ces* operon coding for the emetic toxin synthesis genes is located on a megaplasmid in a restricted range of Bc s.s. strains [7]. More rarely, Bc can also be responsible for extra-digestive pathologies such as ocular or nosocomial infections [8,9]. Although the aforementioned toxins play a crucial role in food poisoning events, Bc also carries many other virulence genes that can modulate its pathogenicity [10].

Unlike the other members of the Bc group, Bt species were initially defined by a capacity to produce parasporal crystals containing toxins, some of which are extremely toxic for insect larvae [11]. Bt strains are therefore widely used as biopesticides in organic and conventional agriculture [12]. These crystals are composed of Cry and Cyt protoxins (also called δ-endotoxins) for which several hundred different haplotypes have been described so far [13]. Their classification has been recently revised based on protein sequence identity [14]. Upon ingestion of spores and crystals by larvae, larval digestive enzymes allow the release and activation of toxins from the crystals. Cry/Cyt toxins bind to specific receptors of the host midgut, forming pores in the cytoplasmic membrane of enterocytes, thereby triggering their death and creating breaches in the intestinal lining. In parallel, the favorable environment of the midgut supports the germination of spores and their entry in the internal milieu [15]. Vegetative bacteria can proliferate, ultimately leading to septicemia and the death of the host. Four subspecies of Bt are commonly used in commercial products. Bt ssp. *aizawai* (Bta) and Bt ssp. *kurstaki* (Btk) target Lepidoptera, Bt ssp. *morrisoni* (Btm) targets Coleoptera and Bt ssp. *israelensis* (Bti) is used against mosquitoes. Due to the ability of Bt to produce a large spectrum of insecticidal molecules, it is now considered as the leading microbial pesticide worldwide [16].

Interestingly, Bc and Bt share some virulence genes, particularly those encoding enterotoxins [6]. In addition, bacterial spores, in particular Bt spores, have the ability to persist in the environment, and have been found on vegetables that have been treated with commercial products [17,18,19,20]. Because Bc s.s. and Bt are genetically very closely related, their distinction is not straightforward, and very few accurate tests are available to address this issue, leading to some authors to propose the merging of Bc s.s. and Bt species several decades ago [13]. Nevertheless, the production of the parasporal crystals, consensually accepted as the primary definition of Bt, can be observed using optical microscopy (NF EN ISO 7932/Amd1), although this method requires expertise and several days to obtain an accurate identification. Based on this approach, retrospective analyses in Canada [21] and France [17] identified Bt in FBOs that were initially attributed to Bc. In a report dealing with the possible involvement of Bt in foodborne infection events, EFSA called for the development of a simple method to differentiate Bt from other Bc members [22], and a fortiori, the Bt strains used as pesticides. In addition, the issue of food monitoring and, in particular, the acceptable dose of Bc/Bt in food is regularly examined by health authorities. The development of new tools for the specific identification of Bt strains would facilitate and extend this monitoring, to prevent and limit the emergence of new intoxication cases.

Genetic proximity between Bt and other members of the Bc group, especially Bc s.s. [2,3], makes it difficult to develop identification methods; however, some genomic approaches may offer a solution. With the development of high-throughput sequencing (HTS), the rapid acquisition of complete genomes has been greatly facilitated. Based on pangenome analyses, recent studies have split the Bc group into three to five major clades [2,23], whereas a previous classification defined seven phylogenetic *panC* groups named I to VII and associated with different levels of psychrotolerance [24]. This phylogenetic group assignment, as well as other typing methods (search for virulence genes and MLST, for instance) have allowed the development of a new computational classification tool for Bc genomes [25]. According to the literature, the entire Bt species is spread across several of these clades or groups, and this taxon should be considered as a polyphyletic biovar, defined by the presence of plasmid genes encoding insecticidal toxins (*cry* and *cyt*) [23,26]. One study has even also proposed a new pairwise ANI-based classification of the Bc group into genomospecies, Bt genomes being mostly (though not completely) grouped with Bc s.s. within the genomospecies of the same name [26].

Most Bt strains of interest with regard to food safety (e.g., isolated from pesticides or FBOs) have been carefully studied and have shown very little divergence from each other and from other Bc strains of phylogenetic group IV, clade 2 [17]. In an attempt to distinguish among them, four proximity clusters (named a to d), composed of FBO-associated Bt and commercial Bta and Btk, have been defined using an SNP calling approach. The clusters have been circumscribed from phylogenetic reconstruction and pairwise single-nucleotide polymorphism (SNP) distance calculations (ranging from 0 to 10 within a cluster and with inter-group distances significantly higher than intra-group SNP distances for each cluster).

The genome-wide association study (GWAS) approach, originally developed for human studies, has been adapted for the analysis of microorganisms [27]. The GWAS approach can establish a statistical link between a feature polymorphism (named hereafter “trait”) and a molecular marker, such as a gene, a k-mer or an SNP. Several studies have already been carried out to correlate the presence of associated markers with a given phenotype, for example, in the context of host-pathogen interactions [28], persistence and resistance to antibiotics [29] as well as low-temperature growth phenotypes in food production [30]. Nevertheless, to date, no studies have identified markers specific to bacterial species or subspecies for the investigation on FBO etiological agents.

In this study, we searched for specific marker sequences allowing the identification of Bt and some Bt groups of interest, among other Bc. We performed a pan-GWAS analysis on 230 complete genomes. A total of 249 genomic markers strongly associated with various traits (Bt species, Bt subspecies and Bt clusters a to d) were identified. Among them, 128 showed potential informativeness as genomic markers after a TBLASTN validation step. Although the Bt species is defined by the presence of genes encoding Cry/Cyt toxins, the identified markers are not related to these genes, due to the high diversity of Cry and Cyt families within the species. For each designated trait, we discovered one marker or a combination of up to six markers able to identify all the Bt in the study dataset. We wrote a Python script using 14 markers for the automated identification of Bt from a genomic assembly.

## 2. Materials and Methods

### 2.1. Whole-Genome Sequencing

The genomic DNA of 78 Bt/Bc isolates was extracted and sequenced as described in Bonis et al. [17]. Briefly, the KingFisher Cell and Tissue DNA kit (Thermo Fisher Scientific, Waltham, MA, USA) was used to isolate genomic DNA. DNA purity and concentration were determined using a Nanodrop Spectrophometer and a Qubit fluorimeter, respectively. Global DNA integrity was visualized on a 0.8% agarose gel (Seakem GTG™ Agarose) after migration for 2 h at 90 V. Library preparation was carried out using the Nextera XT DNA Library Prep kit (Illumina, San Diego, CA, USA) and 150 bp paired-end sequencing of isolated DNA was performed by the *Institut du Cerveau et de la Moëlle epinière*, using a Nextseq500 sequencing system (Illumina). An in-house workflow called ARtWORK v1.0 [17,31] was used to assemble reads with default parameters. The paired-end reads of the isolates are available in the PRJNA781790 BioProject on NCBI and the associated accession numbers are listed in Supplementary Table S1.

### 2.2. Dataset Definition

A total of 286 genomes were used, corresponding to 216 FBO-associated Bc isolates, 18 commercial Bt strains [17], one Bt reference strain, and 51 Bc isolates of various origins associated with robust metadata, whose complete genomes were retrieved from the NCBI RefSeq database (Supplementary Table S1). In addition to the detection of parasporal insecticidal crystals under a microscope (NF EN ISO 7932/Amd1), Bt membership was confirmed by the presence of insecticidal protein-coding genes using the BtToxins_Digger pipeline v1.0.5 [32]. For Bt genomes collected from NCBI, only BtToxins_Digger was used to confirm Bt membership. Using R v4.0.1, a random draw was carried out to split the whole dataset into a study dataset (SD) and a validation dataset (VD) (Supplementary Table S1), representing 80% (*n* = 230) and 20% (*n* = 56) of the whole dataset, respectively. The SD was used as an input for the GWAS analysis, and the VD was used to validate the selected genetic markers. The SD included the genomes of 144 strains belonging to Bt species (i.e., carrying genes encoding crystal proteins), especially from the subspecies *aizawai* (*n* = 56) and *kurstaki* (*n* = 57), and 86 genomes belonging to other Bc, including at least one assembly for each of the nine distinct representative species of the group (Table 1) and for each of the seven previously defined phylogenetic groups [24] given in Supplementary Table S1. The VD included the genomes of 35 strains (62.5%) belonging to Bt species (with more than four subspecies, including ssp. *aizawai*, *kurstaki*, *israelensis* and *morissoni*) and 21 genomes (37.5%) belonging to other Bc, spread across five distinct phylogenetic groups [24] (Supplementary Table S1).

### 2.3. Pangenome Analysis

#### 2.3.1. Pangenome Construction

**Figure 1:**
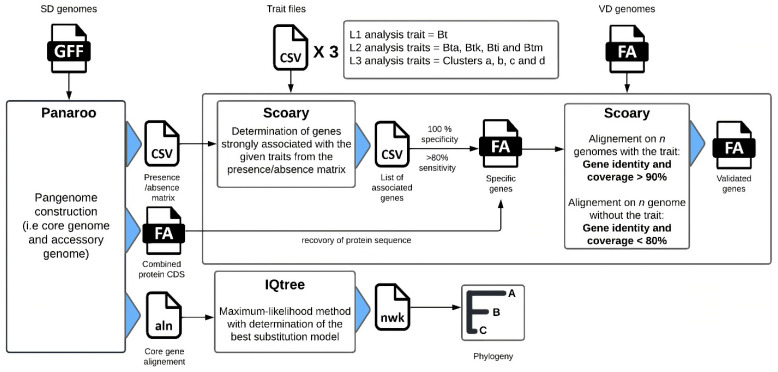
Flowchart of the pan-GWAS analysis. Pangenome construction was first performed using Panaroo [34] to obtain an estimation of the core and accessory genomes of the study dataset (SD). Then, the presence/absence matrix output from Panaroo was used for the GWAS analysis performed using Scoary, thereby providing a list of associated genes for each trait. Only genes with at least 80% sensitivity and 100% specificity for the SD were selected. A TBLASTN validation step of the selected genes was performed after recovering the protein sequences from Panaroo. The genes were validated when the respective sequence criteria of coverage and identity were met and when exhibiting at least 80% sensitivity and 100% specificity for the validation dataset (VD). The core-gene alignment obtained from Panaroo was used to construct the phylogeny of the 230 genomes, using IQtree [36].

An analysis was carried out to deduce the pangenome (i.e., core and accessory genes) from all 230 SD genomes (Figure 1). The whole genome annotation of the assembly was performed using Prokka v1.13.3 [33] with default parameters, and GFF3 files were provided to Panaroo v1.2.3 [34] as input. Panaroo was run using the strict mode with default identity parameters and length difference thresholds (98%), for initial clustering of protein sequences with CD-HIT v4.8.1 [35]. Then, close clusters were collapsed into putative families when they had at least 70% sequence identity. The assignment of a gene cluster to a core genome was defined by the presence of the gene in at least 99% of the genomes (*n* = 228). As output, Panaroo produced a presence/absence matrix, used for the pan-GWAS analysis.

#### 2.3.2. Genotype Association

A three-level pan-GWAS analysis was performed to associate the presence or absence of a gene with a particular phenotype or trait. The first GWAS analysis (named L1 for Level 1) was conducted to search for genes specific to Bt species and the second analysis (named L2 for Level 2) for the Bt subspecies: Bt ssp. *aizawai* (Bta), Bt ssp. *kurstaki* (Btk), Bt ssp. *israelensis* (Bti) and Bt ssp. *morrisoni* (Btm). Then, specific genes for the SNP proximity clusters a, b, c and d within Bt subspecies (a, b for Bta and c, d for Btk) were investigated (analysis L3 for Level 3). These clusters were identified in a previous SNP study [17] and involve Bta or Btk strains that show strong genetic proximity with commercial strains used as insecticides. After targeting traits for each GWAS analysis, a gene search was carried out on the SD. Scoary v1.6.16 [37] was run to identify statistically robust genes (Fisher’s test) associated with each of the designated traits, based on the null hypothesis that the presence/absence of the gene is unrelated to the trait. For each gene, 1000 replicate permutations were conducted and *p*-values were adjusted by applying the Bonferroni correction [38] and the Benjamini-Hochberg procedure [39] (Supplementary Table S2). Only genes with *p*-values (naive and corrected) <0.05 (rejection of the null hypothesis, gene significantly associated with the trait), 100% specificity and at least 80% sensitivity for the screened traits were selected for the validation steps. In case of cluster-specific gene investigation, GWAS analysis (L3) was performed only on SD genomes with clear cluster attribution (*n* = 104), due to the impossibility of determining trait-specific genes when performed against all SD genomes. For each selected gene cluster, a protein sequence was extracted and tested as a candidate marker.

#### 2.3.3. Core Genome Phylogeny

Core-genome sequences from Panaroo were aligned using MAFFT v7.471 [40]. A phylogenetic tree of the SD was built with the maximum likelihood method using iQtree v1.6.9 [36], and the best substitution model GTR + F + R10 was determined with ModelFinder [41] on 286 DNA models. The core genome phylogeny was visualized using Phandango v1.3.0 [42], alongside pangenome metadata. Selected marker presence/absence information within the SD was added to the phylogenetic tree using iTol v6.1.2 [43].

#### 2.3.4. In Silico Validation of Genetic Markers

To certify the reliability of the selected markers, we carried out a TBLASTN v2.7.1+ against the VD to validate candidate marker sequences meeting the required conditions. For each cluster associated with a given marker, a protein sequence was retrieved from the Panaroo output and screened for in the VD genomes. Markers were selected only when sequence coverage and identity were strictly higher than 90% compared with genomes displaying the same trait (intragroup), and when the sequence coverage and identity were strictly lower than 80% compared with genomes not associated with the specific trait (intergroup). This extra step made it possible to test the pan-GWAS selected markers in silico against a new dataset of genomes, and to confirm the sensitivity and the specificity of the markers. To enhance the identification of a genotype, a combination of validated markers associated with the best sensitivity was defined. Predicted genomic localization (i.e., chromosome vs. plasmid) of the gene encoding for the protein selected as a marker was carried out with TBLASTN, using protein sequences and a Bt plasmid database (*n* = 342) collected from PLSBD [44].

## 3. Results

Among the 286 genomes used for this study, 235 (the “ANSES collection”) were sequenced and assembled according to satisfactory quality criteria (median N50: 5,727,322; median number of contig: 103), (Supplementary Table S1). This panel was completed with 51 public genomes from NCBI, associated with the full representation of the genome.

### 3.1. Bacillus cereus Group Pangenome

**Figure 2:**
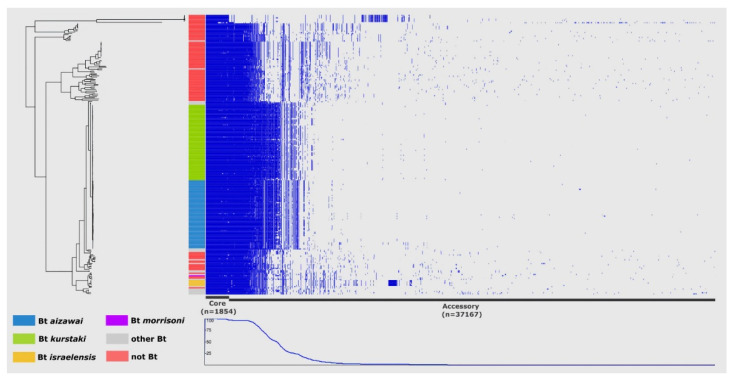
Phylogenetic tree of 230 *Bacillus cereus* (Bc) genomes alongside the gene presence–absence variation matrix. The maximum-likelihood phylogeny (GTR + F + R10 substitution model) was performed with IQtree [36] using the Panaroo [34] core–gene alignment of the study dataset (SD) genomes and visualized with Phandango [42]. The presence–absence matrix obtained with Panaroo identified 1854 core genes (present in 99% of SD genomes) and 37,167 accessory genes for the 230 Bc genomes.

The Bt species is defined here by the presence of Cry/Cyt toxin encoding genes, leading to the release of insecticidal crystals during sporulation. To identify specific Bt markers associated with different traits of interest (Bt species, Bt subspecies Bta, Btk, Bti and Btm, and Bt clusters a to d) using a pan-GWAS approach with Scoary, the pangenome of the Bc group was inferred using Panaroo on SD, resulting in a total of 39,021 clusters of genes. The core genome of the 230 Bc complete genomes was composed of 1854 genes (representing about 5% of all genes), leaving 37,167 accessory genes (Figure 2). Among the accessory genes, 1695 were considered soft core genes (present in 95 to 99% of all genomes), 4358 shell genes (present in 15 to 95% of all genomes) and 31,114 cloud genes (present in less than 15% of all the genomes). A rarefaction curve of the 230 genomes (Supplementary Figure S1) suggests the existence of an open pangenome, as previously assumed for Bc [23,45]. The presence–absence matrix showed that the *B. cytotoxicus* population, which roots the tree, seemed to possess a reduced number of accessory genes. In contrast, the Bta and Btk subspecies, whose genomes represent highly conserved populations, displayed a greater number of accessory genes.

### 3.2. Pan-GWAS Analysis

#### 3.2.1. Gene-Based GWAS

We used Scoary to associate specific genes with the presence or absence of specific traits (Bt species, Bt subspecies Bta, Btk, Bti and Btm, and Bt clusters a to d) with a GWAS analysis. Three GWAS analyses were run to identify a large number of candidate genes for each trait (Table 2). A total of 1304 genes were significantly associated with the traits of interest. Interestingly, the majority of the identified genes were associated with subspecies *israelensis* and *morrisoni* in the GWAS L2 analysis, with 809 and 246 genes, respectively. However, this large number of associated genes can be explained by the low number of genomes available for these two subspecies in the dataset reducing the analytical strength. Given the low diversity in the dataset for these subspecies, we did not pursue the search for specific markers for these subspecies.

Among the other 249 candidate genes, 131 genes met the specificity and sensitivity requirements for the L1 and L2 GWAS analyses, corresponding, respectively, to the Bt species trait and the Bt subspecies traits of the two remaining subspecies (i.e., Btk and Bta). Accordingly, 99 genes were specific to the Bt species and 27 to the Bta subspecies. Interestingly, only 5 genes were specific to Btk, suggesting relatively high diversity within the Btk subspecies. In the L3 GWAS analysis performed on a smaller panel of genomes, 118 genes were identified among the four traits corresponding to clusters a, b, c and d with 10, 14, 46 and 48 genes, respectively.

#### 3.2.2. Validation of GWAS Results

We then validated the markers selected using GWAS by comparing them in silico with the VD using TBLASTN. For each selected candidate gene associated with a specific trait in the GWAS analysis, a protein sequence was extracted and compared to all VD genomes that shared or did not share the trait in question. Out of the 249 highly associated genes obtained with Scoary, 128 protein sequences met the criteria of coverage, specificity and sensitivity of the TBLASTN analysis (Table 2). All validated marker sequences had a sensitivity range between 80% and 100%, and 100% specificity on both SD and VD datasets. When a single marker was not sufficient, combinations of markers were tested to identify all the associated genomes for each trait.

#### 3.2.3. Functions and Distribution of Specific Markers

To predict whether the genes coding for the markers were localized or not on a plasmid, a TBLASTN alignment on a Bt plasmid database was carried out. When the function was known, the closest protein identified in Uniprot [46] was indicated (Supplementary Table S2). Table 3 lists 14 markers associated with low *p*-values, and whose combination could detect all genomes of a given trait (i.e., 100% sensitivity). The Bt species can thus be identified with a combination of only six markers (*cwlA*, *intQ*, group_3916, group_20749, group_20361 and *sdpR*). For example, the *cwlA* gene located in an operon along with *cwlB*, encodes a peptidoglycan hydrolase known for its role in cell lysis during Bt spore release [47]. Regarding the markers that emerged from the GWAS L2 analysis for Bt subspecies, all Bta genomes (*n* = 56) could be identified based on the *apr* gene, which encodes a specific chromosomal alkaline serine protease, also called subtilisin, in *Bacillus subtilis* [48]. For Btk, two genes encoding hypothetical proteins (group_27293 and group_27336) were needed to identify the 57 genomes present in the SD. Moreover, both genes were located on plasmids, suggesting the presence of a common plasmid in these subspecies. Regarding the cluster-specific markers identified with the GWAS L3 analysis, clusters a, c and d could each be identified with a single chromosomal marker, respectively with group_10114 encoding a hypothetical protein, the repressor *lexA,* involved in the SOS system [49], and the gene encoding a subunit of Clp protease [50]. Cluster b, like Btk, needed two markers for the identification of all genomes: the *rapF* gene, involved in bacterial competence [51], combined with a plasmid gene of unknown function (group_20667). For all hypothetical protein-coding genes, we attempted to annotate them with the 3D structure using SWISS-MODEL [52]. However, it was not possible to assign new putative functions to them (Supplementary Table S3).

**Figure 3:**
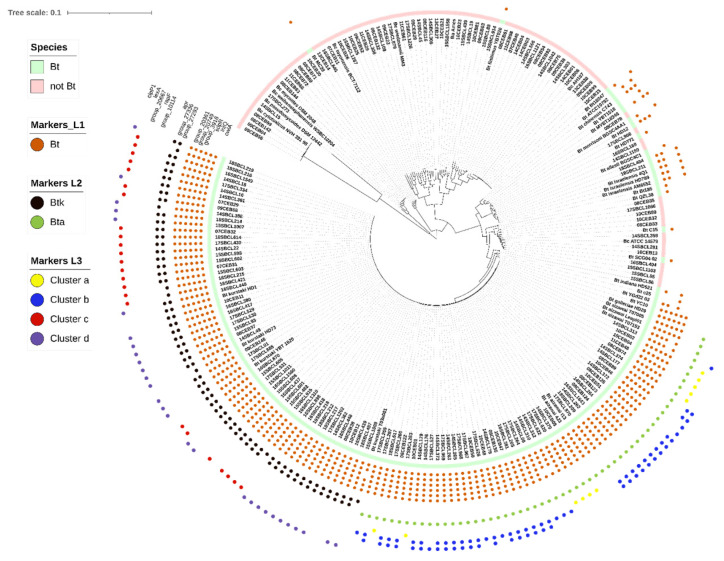
Core genome phylogeny of 230 *Bacillus cereus* (Bc) genomes. The maximum-likelihood phylogeny (GTR + F + R10 substitution model) was performed with IQtree [36] using a Panaroo [34] core–gene alignment of the study dataset (SD) genomes. Visualization and annotation were performed using iTol [43]. Filled circles indicate the presence of the 14 Bt markers for the successive GWAS analysis levels (L1, L2 and L3) in the corresponding SD genomes.

Phylogenetic reconstruction of the 1854 core genes from the 230 genomes (Figure 3) showed a clear distinction between most Bc genomes, including Bt genomes, and five genomes of the *B. cytotoxicus* species, which is known to be the most distant group member [2]. In contrast, the genomes of the two subspecies Btk and Bta were located on the outermost branch of the tree compared with *B. cytotoxicus* genomes. Unsurprisingly, other Bt subspecies or unknown subspecies genomes were scattered around the tree. Some of them, such as MTC28 (NCBI accession: NC_018693.1) and Bt *finitimus* YBT020 (NCBI accession: NC_017200.1) were phylogenetically very distant.

The 14 Bt-specific markers identified with the three levels of GWAS analysis and validated by TBLASTN were mapped on the phylogenetic tree (Figure 3). Despite the great distance between some Bt strains, a combination of six markers can identify all of them. Similarly, with two markers specific to Btk and cluster b, all associated genomes can be retrieved. Only one marker was needed for the identification of Bta, clusters a, c and d.

#### 3.2.4. Workflow for Bt Identification

**Figure 4:**
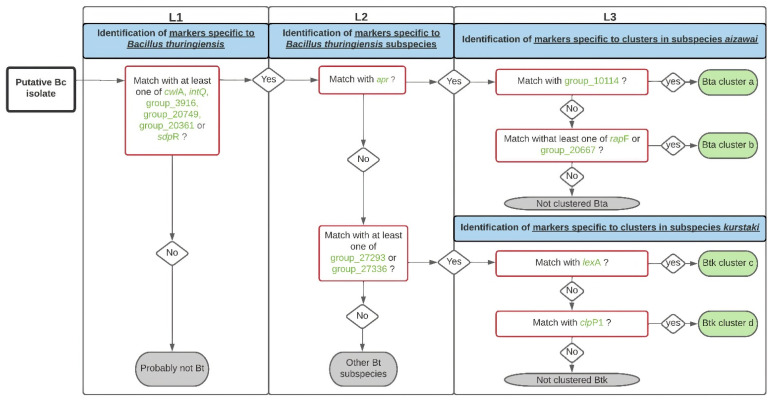
Workflow for *Bacillus thuringiensis* (Bt) identification. The proposed decision tree is used to determine if a putative *Bacillus cereus* (Bc) isolate belongs to the Bt species (L1), to the Bt ssp. *kurstaki* (Btk) or *aizawai* (Bta) subspecies (L2) and to the clusters a, b, c, and d (L3), based on the 14 marker protein sequences previously identified (see Table 3). A marker is considered present when its sequence coverage and its identity are both higher than 90% in the assembly tested.

Based on the 14 selected markers, a workflow was developed to distinguish Bt from the genome of a putative Bc isolate (Figure 4). We wrote an in-house script in Python 3 to automate the screening of the markers in genome assemblies. Using BLASTX, the script detects the markers for Bt, Bt subspecies and Bt clusters using the same thresholds as the TBLASTN validation (90% identity and coverage) in the user-input genomes. The output indicates the presence (+) or absence (−) of all trait-associated markers for each genome query. Then, according to the workflow pathway, based on the presence/absence of the markers within the genome, a putative Bt identification is given. The script is freely available at https://github.com/afelten-Anses/Bt_typing (accessed on 2 February 2022).

## 4. Discussion

To differentiate Bt from other members of the Bc group and especially in the context of food poisoning, we analyzed a large database of Bc to identify genetic markers specific to Bt. The construction of the Bc pangenome from 230 Bc genomes showed that it was composed of 39,021 protein-coding genes and that the core genome corresponded to 1854 genes. These results differ somewhat from those previously published for the Bc pangenome. A previous pangenome study of 114 Bc highlighted 59,989 genes; however, only 600 were described as core genes [23]. Normally, increasing the number of genomes in a dataset can lead to a decrease in the core genome size, along with an increase in the accessory genome size. However, the difference in tools used for pangenome deduction here can explain this large difference in gene numbers, as already highlighted in a genome-wide analysis of *Clostridium difficile* pangenome [53]. For instance, the previous popular pangenome analysis software, named Roary [54], uses a default threshold of 95% identity (versus 98% with Panaroo) to create clustered protein sequences. Nonetheless, Panaroo, unlike Roary, clusters potential close family genes together. Moreover, Roary does not take assembly and annotation errors into account, thereby resulting in an overestimation of the total gene number and thus a reduction of the core genome size, especially for datasets with high diversity as in the Bc group.

In addition, many Bt genomes (especially from the Btk and Bta subspecies) that were used for this study comprised a large number of accessory genes, which can be explained by the high diversity of the genomes in these subspecies in the dataset, and/or the presence of mobile genetic elements, particularly plasmids. Several studies have already highlighted that the number of plasmids is high in Bt, in comparison with Bc [55,56,57]. A comparative genomic analysis study has shown that Bt strains with high insecticidal potency harbor genes promoting infection, immune evasion and nutrient access that may play a key role in entomopathogenicity and host adaptation [58]. Toxin-carrying plasmids, which represent an important part of the Bt plasmid pool, have also been shown to be involved in cellular functions such as germination, sporulation and horizontal gene transfer [55,59]. For the Bti and Btm subspecies, for which only a limited number of genomes were included in this study, no gene validation step could be performed to define specific markers. Nevertheless, our analysis led to the identification of genes only present in a restricted population of Bti (18SBCL211A, 18SBCL484A, Bt_israelensis_4Q1, Bt_israelensis_HD789, Bt_israelensis_AM6552) or in one strain of Btm (Bt_morrisoni_BGSC 4AA1), whose specificity deserves further investigation.

A total of 249 candidate genes were selected based on their sensitivity and specificity for a given trait. As recommended for GWAS analyses [60], the results obtained were validated by testing them on a test dataset (here, the VD). The fact that almost 50% of the selected markers did not pass this step demonstrates the importance of testing results. To extend identification to other traits or Bt genomes, marker selection preferentially targeted genes with a chromosomal location and a known or predicted function. Of the 128 validated genes, only 21 had predicted functions, annotated with Prokka [33] (Supplementary Table S3). At least one gene associated with a known function and located on the chromosome was identified for L1- and L2-associated traits (Bt species and subspecies, respectively), with the exception of Btk. However, we showed that Btk could be identified based on the combination of two plasmid markers, which underlines the importance of plasmid content, especially for the discrimination of closely related Bt strains.

The phylogenic visualization of the SD dataset illustrates the challenge—even when using genomic approaches—of differentiating Bt populations from each other or other Bc group members, due to their close genetic proximity [3,23]. For example, L3 analysis performed for cluster identification could not be performed on the whole SD. The impossibility of identifying specific genes revealed a limitation of gene-based GWAS when comparing extremely close groups. Fortunately, the sequential use of a combination of markers allows the use of cluster-specific markers after confirmation of Bt membership and then one of two subspecies, Bta or Btk. Furthermore, the use of a highly diverse dataset in this study allowed the identification of specific and high sensitivity markers. For example, among the Bt markers identified, the *cwlA* gene was found in 93.8% of SD Bt genomes (*n* = 144), making it a very reliable marker compared with the previously proposed detection system [61,62,63]. Furthermore, this previous system did not include commercial Bt strains, unlike the analyses performed for this study. Nevertheless, it is noteworthy that the identified markers refer to a specific dataset, meaning they may not allow the identification of the entire Bt species. In addition, we cannot exclude the existence of some non-sequenced strains, in particular divergent strains not included in our dataset that may not possess one or more of the selected markers. However, the workflow developed here can easily be adapted with the addition of new markers or modification of existing markers to detect additional Bt strains of interest.

The 2016 EFSA report [22] highlighted the need for the development of new methods to differentiate among Bc members, in particular to discriminate Bt from Bc s.s.. An interesting tool (BTyper3) has been developed based on a compilation of typing methods from sequencing data (including typing virulence genes), for the classification of Bc members, in particular the virulent *B. anthracis* and emetic strains [25]. The markers we highlighted here constitute a complementary approach, because they allow the accurate detection of Bt strains of interest for food safety. To differentiate Bt from other members of the Bc group, particularly in the context of FBOs, our work shows that a combination of six markers can identify Bt species, three for the two major subspecies of interest in food safety, and five for the proximity clusters of pesticide strains frequently used in agriculture. Based on these 14 markers, we developed a new tool, associated with a workflow (Figure 4) and a script to predict the identity putative Bt among Bc isolates. Moreover, the gene-based GWAS approach developed here demonstrated that the four proximity clusters associated with commercial Bta or Btk strains likely belong to Bt [17]. Currently, the workflow cannot be used to distinguish certain Bt strains (particularly the insecticide strains belonging to the *kurstaki* subspecies) within the same cluster. Gene-based approaches are limited in their specific identification of commercial Bt strains used in agriculture. New approaches, notably based on SNP calling, can be conducted to search for differentiation methods at the strain level. In this perspective, our workflow can be easily updated and optimized, if necessary.

With the routine use of sequencing methods in laboratories and the significant development of high-throughput sequencing techniques, the use of computational tools as a complementary method for the identification of bacterial species may prove to be a valuable asset, especially in the context of food poisoning. This complementary identification method can be used to quickly assign a Bc strain to the Bt species, as well as to challenge false positives and false negatives resulting from microscope searches for protein crystals, possibly due to ambiguous phenotypes, misinterpretation of results, or loss of plasmids carrying the *cry* genes. With different levels of analysis, possible assumptions on the origin of the isolates can be made.

## 5. Conclusions

Bt biopesticides constitute a topic of concern in food safety due to their wide use in agriculture and their suspected association with food poisoning events, therefore warranting the development of tools for their traceability in food. This study identified specific molecular markers, usable either alone or in combination, for the detection of the most widely used Bt pesticides, at the scale of the Bt species, subspecies (*aizawai* and *kurstaki*) and four clusters of genetic proximity (a to d) previously defined [17] referring to seven Bt pesticidal strains. Thus, each presumptive Bc genome can be classified according to an established workflow into Bt species (versus non-Bt, *n* = 6 markers), then into subspecies (*kurstaki n* = 2 markers, *aizawai n* = 1 marker), and finally into genomic proximity clusters (a: *n* = 1 marker, b: *n* = 2 markers, c: *n* = 1 marker and d: *n* = 1 marker). A command line tool, based on a 14-marker workflow, was developed for the automated serial search of these markers (https://github.com/afelten-Anses/Bt_typing, accessed on 2 February 2022).

## Figures and Tables

**Table 1:** Composition of the study dataset (SD).

Species	Subspecies	No. of Genome(s)	Representative Genome	NCBI Accession Number
*Bacillus anthracis*		1	Ames	NC_003997.3
*B. cereus (sensu stricto)* (Bc s.s.)		2	ATCC 14579	NC_004722.1
*B. cytotoxicus*		1	NVH 391-98	NC_009674.1
*B. mycoides*		1	DSM 2048	NZ_CM000742.1
*B. pseudomycoides*		1	DSM 12442	NZ_CM000745.1
*B. thuringiensis* (Bt)	Bt ssp. *aizawai*	56	Leapi01	AMXS00000000.2
	Bt ssp. *kurstaki*	57	HD-1	NZ_CP004870.1
	Bt ssp. *israelensis*	5	AM6552	NZ_CP013275.1
	Bt ssp. *morrisoni*	1	BGSC 4AA1	NZ_CP010577.1
	other and/or unknown	25	ATCC 10792	NZ_CP021061.1
*B. toyonensis*		1	BCT-7112	NC_022781.1
*B. weihenstephanensis*		1	WSBC 10204	NZ_CP009746.1
*B. wiedmannii*		1	MM3	NZ_CM000718.1
other *B. cereus (sensu lato)* *		77		

* genomes of Bc isolates collected from foodborne outbreaks, and for which, under sporulation conditions, no crystals were detected using optical microscopy.

**Table 2:** Summary results of the validation of specific markers associated with three levels of GWAS analysis (L1, L2 and L3).

\	Scoary				TBLASTN
GWAS Analysis	Trait	No. of Genome(s) Associated with Trait	No. of Genome(s) Exempt from Trait	No. of Genes Strongly Associated with Trait *	No. of Genes Validated In Silico (Annotated)
L1 (*n* = 230)	*Bacillus thuringiensis* (Bt)	144	86	99	32 (22)
L2 (*n* = 230)	Bt ssp. *aizawai*	56	169	27	17 (4)
	Bt ssp. *kurstaki*	57	168	5	3 (0)
	Bt ssp. *israelensis*	5	220	809	NA (78)
	Bt ssp. *morrisoni*	1	224	246	NA (101)
L3 (*n* = 104)	Cluster a	11	93	10	6 (0)
	Cluster b	40	64	14	10 (1)
	Cluster c	24	80	46	30 (1)
	Cluster d	29	75	48	30 (3)

Level 1 analysis (L1) refers to markers associated with the Bt species; level 2 analysis (L2) refers to markers associated with the Bt subspecies; and level 3 analysis (L3) refers to markers associated with the proximity clusters a, b, c and d, within Bt subspecies *aizawai* and *kurtaki* (Bla and Btk). * genes with a low *p*-value (<0.05) in the Scoary analysis; at least 80% sensitivity and 100% specificity for genomes associated with the trait in question.

**Table 3:** List of 14 genes with strong evidence and best combinations for *Bacillus thuringiensis* (Bt) trait identification associated with three GWAS analysis.

GWAS Analysis	Gene Name	Replicon	Annotation	UniprotKB	Trait	Marker Sensitivity (%)	Cumulative Sensitivity (%)	*p*-Value *	NCBI Accession Number
L1	*cwlA*	Chromosome	N-acetylmuramoyl-L-alanine amidase CwlA	P24808	Bt	93.8	93.8	1.72 × 10^−51^	WP_021728236.1
	*intQ*	Chromosome	Putative defective protein IntQ	P76168	Bt	91.7	94.4	1.64 × 10^−48^	WP_000237488.1
	group_3916	Chromosome	Hypothetical protein	N/A	Bt	88.9	95.1	3.29 × 10^−47^	WP_000858032.1
	group_20749	Chromosome	Hypothetical protein	N/A	Bt	86.8	97.2	6.36 × 10^−45^	WP_042596929.1
	group_20361	Plasmid	Hypothetical protein	N/A	Bt	84.7	98.6	8.43 × 10^−43^	WP_002101540.1
	*sdpR*	Plasmid	Transcriptional repressor SdpR	O32242	Bt	84.0	100.0	4.00 × 10^−42^	WP_000998670.1
L2	*apr*	Chromosome	Subtilisin	P04189	Bta	100.0	N/A	9.10 × 10^−53^	WP_021728520.1
	group_27293	Plasmid	Hypothetical protein	N/A	Btk	98.2	98.2	5.83 × 10^−52^	WP_003273526.1
	group_27336	Plasmid	Hypothetical protein	N/A	Btk	89.5	100.0	1.05 × 10^−43^	WP_001293418.1
L3	group_10114	Chromosome	Hypothetical protein	N/A	Cluster a	100.0	N/A	4.48 × 10^−15^	WP_000415284.1
	*rapF*	Chromosome	Response regulator aspartate phosphatase F	P71002	Cluster b	92.5	92.5	4.82 × 10^−25^	WP_050062578.1
	group_20667	Plasmid	Hypothetical protein	N/A	Cluster b	82.5	100.0	1.34 × 10^−20^	WP_131256056.1
	*lexA*	Chromosome	LexA repressor	P31080	Cluster c	100.0	N/A	4.31 × 10^−24^	AHZ54004.1
	*clpP1*	Chromosome	ATP-dependent Clp subunit 1	B0B803	Cluster d	100.0	N/A	2.13 × 10^−26^	WP_000791073.1

* Naive *p*-value provided by Scoary (Fisher’s test). A *p*-value < 0.05 leads to the rejection of the null hypothesis that the presence of the gene is unrelated to the trait; the gene is therefore significantly associated with the trait in question. Level 1 analysis (L1) refers to markers associated with the Bt species; level 2 analysis (L2) refers to markers associated with the Bt subspecies; and level 3 analysis (L3) refers to markers associated with the proximity clusters a, b, c and d, within Bt subspecies *aizawai* and *kurtaki* (Bla and Btk).

## Data Availability

Data are presented in the manuscript and the associated Supplementary Materials. The sequencing data used in this study are available under the two BioProjects: PRJNA547495 and PRJNA781790 on NCBI.

## Supplementary Materials

The following supporting information can be downloaded at: https://www.mdpi.com/article/10.3390/foods11233924/s1, Figure S1. Rarefaction curve of gene diversity as a function of the number of samples generated with non-parametric incidence-based estimator jack1 [64]. Table S1. List of the 286 genomes used in this study. The genome set was divided into two datasets, a study dataset (SD) (*n* = 230) and a validation dataset (VD) (*n* = 56). ^a^ Clusters were determined by SNP calling, using iVARCall2 [17]. ^b^ All the sequencing data used in this study are associated with two BioProjects: PRJNA547495 [17] and PRJNA781790 (this study). The attribution to panC groups was performed after partial sequencing of *panC* gene, and according to Guinebretière et al., [24]. Abbreviations: FBO = foodborne outbreak. Table S2. List of the 21 annotated genes found with GWAS analysis and validated using TBLASTN. Table S3. Search for 3D structure prediction for workflow markers of unknown function. The search was performed using SWISS-PROT. ^a^ GMQE (global model quality estimation) is a quality estimation that combines properties from the target–template alignment and the template structure. ^b^ QMEAN is a composite estimator based on different geometrical properties and provides both global (i.e., for the entire structure) and local (i.e., per residue) absolute quality estimates on the basis of one single model. ^c^ QSQE (quaternary structure quality estimate) score is a number between 0 and 1, reflecting the expected accuracy of the interchain contacts for a model built based on given alignment and template.
