# Supplementary figures and images for: Identification of Genetic Markers for the Detection of Bacillus thuringiensis Strains of Interest for Food Safety
Source: Foods. 2022 Dec 5;11(23):3924. doi: 10.3390/foods11233924 (PMC9739007; doi:10.3390/foods11233924)

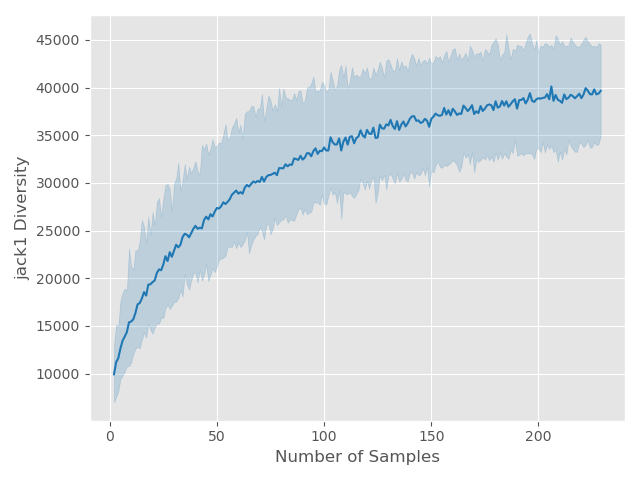

Supplement: Supplementary file 1 [file foods-11-03924-s001.zip › Supplementary Fig S1.jpg]
